# Assessment of Diastolic Function in Congenital Heart Disease

**DOI:** 10.3389/fcvm.2017.00005

**Published:** 2017-02-15

**Authors:** Dilveer Kaur Panesar, Michael Burch

**Affiliations:** ^1^Cardiothoracic Unit, Great Ormond Street Hospital for Children NHS Foundation Trust, London, UK; ^2^Centre for Cardiovascular Imaging, Institute of Cardiovascular Science, University College London, London, UK

**Keywords:** diastolic function, congenital heart disease, echocardiography, CMR, diastolic heart failure

## Abstract

Diastolic function is an important component of left ventricular (LV) function which is often overlooked. It can cause symptoms of heart failure in patients even in the presence of normal systolic function. The parameters used to assess diastolic function often measure flow and are affected by the loading conditions of the heart. The interpretation of diastolic function in the context of congenital heart disease requires some understanding of the effects of the lesions themselves on these parameters. Individual congenital lesions will be discussed in this paper. Recently, load-independent techniques have led to more accurate measurements of ventricular compliance and remodeling in heart disease. The combination of inflow velocities and tissue Doppler measurements can be used to estimate diastolic function and LV filling pressures. This review focuses on diastolic function and assessment in congenital heart disease.

## Introduction

Myocardial function has been extensively studied in the context of congenital heart disease. The focus to date has been on systolic function due to its importance and ease of measurement. However, the heart also needs to be adequately filled in order to function optimally, and this aspect of cardiac function is relatively under-investigated. The difficulties (as with systolic function) come when attempting to measure the relaxation of the myocardium while negating any effect of pre- or afterload. This is a difficult task and has led to the development of several tools which track myocardial movement independently of the usual flow-based parameters; the latter are heavily influenced by loading conditions. This article will summarize the current assessment of diastolic function using echocardiography and cardiac magnetic resonance (CMR) imaging in the normal heart and in patients with congenital heart disease.

## Diastolic Dysfunction (DD)

Diastole denotes the filling phase of the cardiac cycle. Filling is determined by myocardial relaxation as well as atrial contraction and atrial and ventricular compliance. Myocardial relaxation begins when the myofibrils return to an unstressed state and this precedes mitral valve (MV) opening (isovolumic relaxation). ATP is used to actively uncouple calcium from the contractile apparatus and return it to the sarcoplasmic reticulum. Active relaxation is only responsible for early diastolic filling, whereas compliance is important throughout filling and especially during atrial contraction.

The early part of diastole is active relaxation, which is an energy-consuming process. The latter part is due to compliance or stiffness of the ventricle. Isovolumic relaxation time (IVRT) can be measured by invasive catheterization measurements. The index used in its measurement is the time constant of isovolumic pressure decline (τ). In non-invasive measurement, IVRT is the closest measurement to assess this value. However, as with all indices of diastolic function, the loading conditions must be taken into account.

The stiffness of the myocardium also plays an important role in diastolic function. The mass of the left ventricular (LV) affects the stiffness as do the viscoelastic properties of the myocardium (cellular and extracellular components). Attempts are made to measure this increase in myocardial stiffness. However, the difficulty arises in the mechanism of measurement as well as the timing and nature of diastole. Flow-based measurements rely on a change in volume to occur, and so they are unable to quantify isovolumic relaxation as they assess only the last stage of diastole. There is also no universal measurement of diastole [equivalent to ejection fraction (EF) in systole] and torsion and dyssynchrony are difficult to quantify.

## Non-Invasive Assessment Methods

### Echocardiography

As the ventricle stiffens, the velocity of blood flowing into the ventricle decreases. This downward trend would continue if not for compensation which takes the form of increased heart rate or increased diastolic filling pressure to maintain stroke volume (SV). The latter causes an increase toward normal filling velocities (pseudonormalization) and a prolongation of rapid filling. Ventricular elastance is an important marker for ventricular stiffness and is correlated with increased cardiac morbidity (1). Increased strength of atrial contraction is a related compensatory mechanism to cope with increased diastolic filling pressures.

### Left Atrium (LA) Volume

The LA is exposed to the LV loading pressure during MV opening and gradually remodels and increases in size. As LA remodeling takes place over time, it is a marker of the duration of DD as well as severity. The amount of dilatation also correlates with cardiovascular risk burden (2). There is a correlation between LA size and the risk of developing congestive cardiac failure (3, 4), atrial fibrillation (5), and ischemic heart disease (6). LA size is also a predictor of adverse outcome in patients with hypertrophic cardiomyopathy (7). LA size is measured from the apical four-chamber view at end-systole. It is also possible to calculate LA area from 2D four-chamber and two-chamber views.

### Transmitral Doppler Inflow

**Figure 1:**
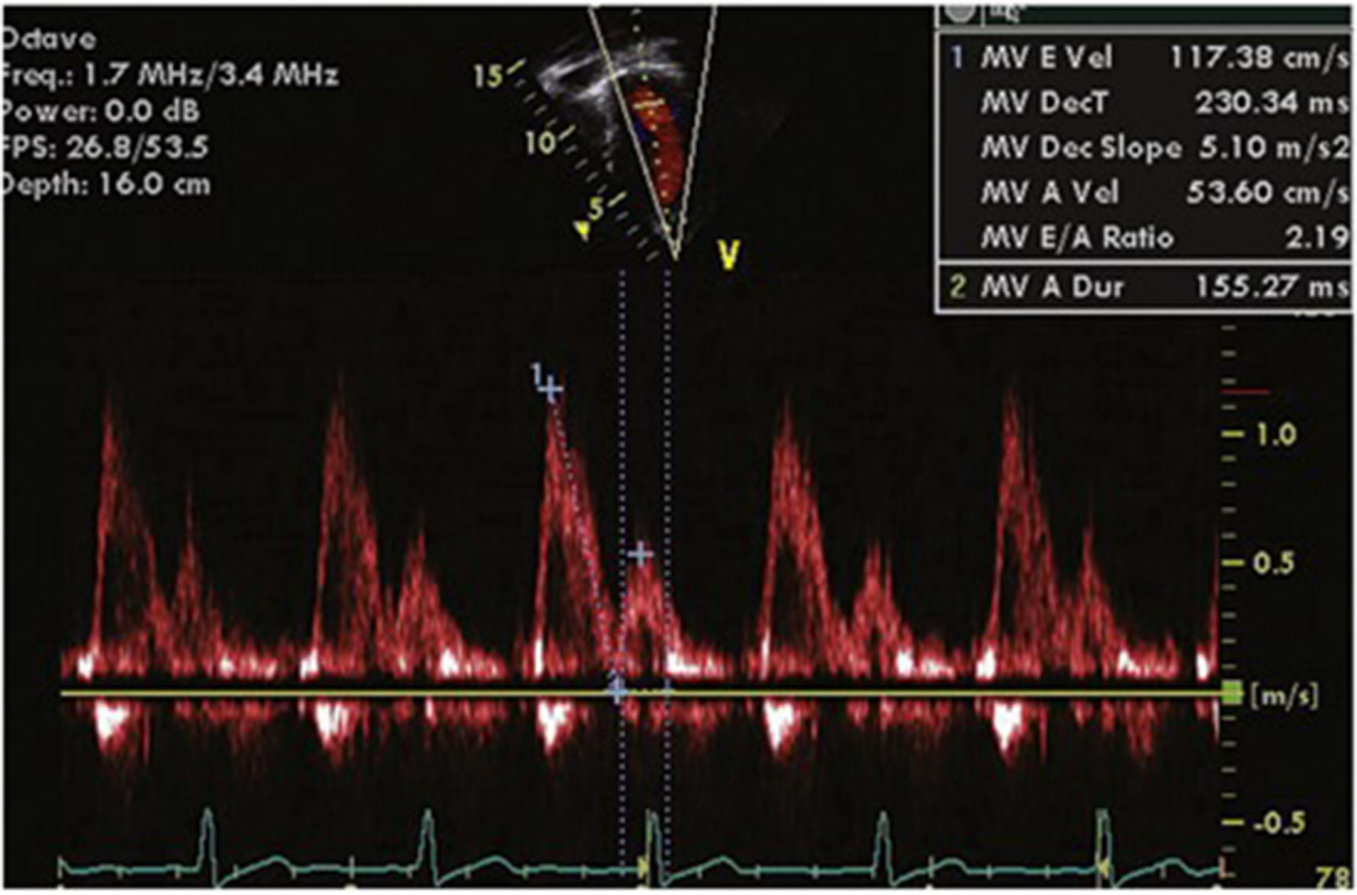
**Normal mitral inflow velocity profile**. Maximum E Velocity (cm/s) = early diastolic mitral inflow velocity. MV Deceleration Time (ms) = duration of deceleration of E wave. MV Dec Slope (m/s^2^) = rate of decrease of E wave. Maximum A Velocity (cm/s) = atrial component of mitral filling. A wave duration (ms) = duration of A wave. MV E/A ratio = ratio of E velocity to A velocity (normal value <8).

The mitral inflow velocity profile helps characterize LV inflow dynamics. It is best measured from the apical four-chamber view (in both children and adults) with the cursor placed across the MV just inside the LV. The E wave is the early diastolic filling wave seen on Doppler interrogation of the MV. It is caused by the drop of LV pressure below LA pressure during the cardiac cycle and is therefore influenced by LA pressure, LV compliance, and the rate of LV relaxation. The A wave, or atrial contraction wave, is immediately after the E wave on Doppler flow analysis. This is influenced by LV compliance and LA pressure and LA contractility rate. All MV inflow velocities are affected by preload and afterload. Under normal conditions, the E velocity is greater than A velocity (Figure 1). As the ventricle becomes less compliant, the E velocity decreases and the ratio lowers. When the A velocity surpasses the E velocity, true DD is present. The mitral inflow is affected by preload, heart rate (including arrhythmias) and age (8).

E wave deceleration time is the rate at which the atrial and ventricular pressures equilibrate after onset of the E wave and is shorter in compliant ventricles (160–240 ms in adults). The IVRT is the period between closure of the aortic valve and opening of the MV. This is normally 70–90 ms long in adults and is prolonged in the case of decreased LV compliance. It is also affected by heart rate and ventricular function. It is best recorded from the apical five-chamber view with the cursor placed to record LV outflow tract velocities and LV inflow simultaneously.

### Pulmonary Venous (PV) Inflow

Pulmonary venous flow can enhance the information provided by MV inflow velocities. The pulsed-waved Doppler cursor should be placed in the right or left upper pulmonary vein from the apical four-chamber view, as distally into the vein as possible. The variables which are measured include peak systolic flow velocity (S), the peak diastolic flow velocity (D), peak atrial reversal flow velocity (AR), and AR duration (ARdur). The normal PV flow profile shows an initial, large S wave followed by a small D wave and then some retrograde flow during atrial contraction. As LA pressures increase, flow becomes predominantly diastolic and the S/D ratio reverses. A decreased systolic fraction of 40% is associated with elevated mean LA pressure of >15 mmHg (9). AR and ARdur also help, as an increase in AR velocity and duration indicates increased LA pressure.

### Color M-Mode Doppler

An intraventricular pressure gradient exists between the base and apex of the ventricle, which acts to cause a suction effect on blood during diastole (10, 11). This can be measured using color M-mode across the MV (from the apical four-chamber view) and measuring the slope of the first aliasing velocity (red–blue) from the MV plane to 4 cm distal in the LV (Vp in centimeters per second) (12). This is known as the color M-mode velocity propagation index (Vp). Vp is not subject to pseudonormalization, which suggests that it is preload independent (13). It does not change with alteration of preload in dogs (14, 15) and humans (14, 16). There is an inverse correlation between the isovolumetric time constant of relaxation (τ) and Vp in humans (13, 14, 17) and dogs (14). Vp is associated with ventricular wall relaxation, becoming less steep as diastolic function worsens (17). The ratio of early LV filling (E) to Vp is a commonly used parameter, which corresponds to pulmonary capillary wedge pressure (PCWP), brain natriuretic peptide, and NYHA class (16).

## Assessment of Severity

**Figure 2:**
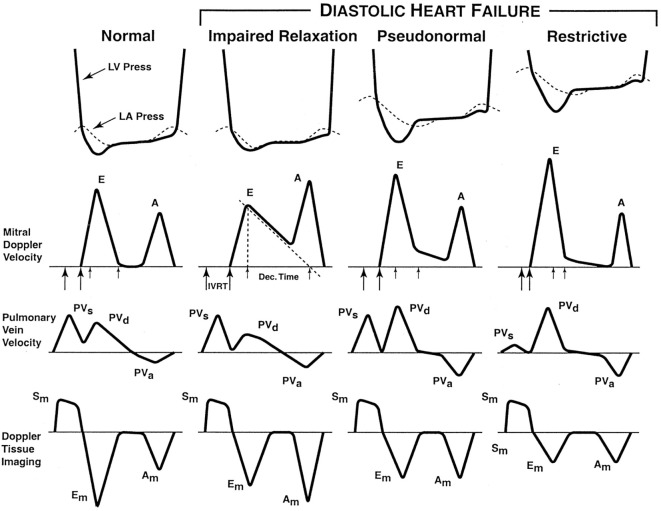
**The stages of diastolic heart failure**. LV and left atrial (LA) pressures during diastole, transmitral Doppler LV inflow velocity, pulmonary vein Doppler velocity, and Doppler tissue velocity. IVRT indicates isovolumic relaxation time; Dec. Time, e-wave deceleration time; E, early LV filling velocity; A, velocity of LV filling contributed by atrial contraction; PVs, systolic pulmonary vein velocity; PVd, diastolic pulmonary vein velocity; PVa, pulmonary vein velocity resulting from atrial contraction; Sm, myocardial velocity during systole; Em, myocardial velocity during early filling; and Am, myocardial velocity during filling produced by atrial contraction.

There are different grades of DD. Early (grade I/impaired relaxation) dysfunction is caused by a decrease in LV compliance, thereby leading to increased LV filling pressure. This delays atrial emptying and prolongs the E wave deceleration time (DT > 240 ms). Atrial contraction becomes more vigorous, reducing the E/A ratio to <0.9. Worsening LV DD leads to increased atrial pressure and a decrease in the pressure gradient between the LA and LV, thereby leading to a shortened DT. The E/A ratio increases (0.9–1.5), but the E/A profile may appear normal (grade II or pseudonormalization). However, the e′ velocity on tissue Doppler imaging (TDI) (see below) remains low, giving a clue to the underlying abnormality (18). In grade III dysfunction (restrictive), the E/A ratio is >2, DT <160 ms, and the inflow profile can be altered by the Valsalva maneuver. This works because LA pressure is reduced during the strain phase of the Valsalva maneuver, and this unmasks the underlying DD. In grade IV dysfunction, the abnormalities are fixed in the face of the Valsalva maneuver, as LA pressure is too elevated to respond to decreased preload (see Figure 2). The timing of onset of E and e′ waves is important. Normally, the e′ occurs at the onset of or before the E wave. If the LA pressure is elevated, the E wave may precede e′ (19, 20).

### Tissue Doppler Imaging

Tissue Doppler imaging directly measures myocardial wall velocities by focusing on the high-amplitude, low-frequency signals reflected by the myocardium rather than the blood pool. The areas sampled include the lateral aspect of the mitral annulus in the apical four-chamber view, the basal septal region in the same view, and the lateral tricuspid valve annulus. This serves to minimize translational artifact and to align the probe with the direction of movement. Three waves are usually seen—the systolic (s′) wave, the early diastolic (e′) wave, and the late diastolic wave caused by atrial contraction (a′). Normal values and *Z*-scores are available for each age group in pediatrics (21).

Nagueh et al. were the first to show that E/e′ ratio (ratio of transmitral E velocity and TDI mitral annular e′ velocity) corresponded to PCWP (18). In 125 patients classified by systolic and diastolic function and symptoms, PCWP correlated strongly with E/e′ ratio *r* = 0.87. PCWP correlated only weakly with E velocity but not e′ velocity. Patients with abnormal relaxation and pseudonormalization of the mitral inflow E/A ratio had a decreased e′ velocity (*P* < 0.001). In patients with DD, a saline bolus affects the E/A wave as measured by transmitral Doppler measurement but did not affect the e′ or e′/a′ ratios (22). These studies show that e′ acts as a preload-independent marker of LV relaxation. E wave velocity on mitral inflow Doppler, corrected for e′, correlates strongly with PCWP, and can be used to estimate LA pressure non-invasively.

Tissue Doppler imaging can also differentiate between restrictive cardiomyopathy and constrictive pericarditis (23). As e′ is a property of the myocardium, theoretically, it should remain unchanged in the presence of extrinsic constriction and should only be reduced in true restrictive cardiomyopathy. This is indeed the case, with a significantly lower e′ in restrictive cardiomyopathy than constrictive pericarditis (*P* < 0.001) (23). This has been corroborated by other groups (24, 25).

E/e′ ratio is an independent predictor of outcome in patients assessed 1–6 days after acute myocardial infarction (26). In a population study of 2,042 patients in the community, any degree of DD was predictive of all-cause mortality, whether the patient had clinical symptoms or not (27). In the ADEPT trial, of 225 patients with symptomatic heart failure (HF), diastolic parameters including shorter deceleration time, lower S/D pulmonary vein flow ratio, and increasing E/e′ and E/Vp ratios were all independent predictors of the primary end-points of death, hospitalization, or transplantation (28).

## Emerging Techniques for Assessment of Diastolic Function

### Torsion

Measurement of LV torsion provides insight into an important mechanism of LV filling and ejection. LV rotation is sensitive to changes in regional and global LV function (29–31). MRI tagging can be used for this purpose but can be difficult and costly to obtain (31–34). Speckle tracking may be used to measure torsion, using the largely experimental practice of “torsion echocardiography” (35–37).

### Strain

Strain is a dimensionless index of change in myocardial length in response to applied force and is expressed as a fraction or percentage change. Strain rate is the change in length over time (per second). By convention, myocardial lengthening or thinning is given a positive value. The techniques used to measure strain include echocardiography M-mode (38) or TDI (39, 40) as well as MRI tagging (41, 42). The limitations of echocardiographic measurement include beam direction, which will allow measurement of longitudinal, radial, and circumferential directions depending on the view and angle of interrogation.

Strain imaging aims to provide a high-resolution, real-time measure of myocardial deformation which is independent of loading conditions. A normal pattern of diastolic relaxation has been studied and described (43). Strain imaging can be used to distinguish between restrictive cardiomyopathy and constrictive pericarditis (44) as well as physiological hypertrophy and hypertrophic cardiomyopathy (45).

In a study of 194 patients with chronic systolic HF, global longitudinal strain (GLS) correlated with worse NYHA class and higher NT-proBNP. It also correlated with LV structure and LVEF, as well as LV and RV DD. GLS also predicted long-term adverse events after adjustment for age, ischemic etiology, E/e′ septal, and NT-proBNP with HR 2.04 (*P* = 0.024).

## CMR Imaging

Magnetic resonance imaging can be used to assess diastolic function by the inflow of blood or the movement of myocardium in much the same way as echocardiography. A number of techniques exist which can help to evaluate diastolic function: gradient echo assesses functional dimensions; phase-contrast measures flow, and myocardial tagging measures regional dynamics (46). Flow measurements are more complete as they include the entire annulus, rather than a point as in echocardiography. Tissue phase mapping (TPM) can be used to measure myocardial velocities and obtain similar information to TDI. This process allows calculation of E/Ea ratio (E = MV inflow E wave and Ea = early myocardial relaxation on TPM).

It is also possible to evaluate tissue characteristics (47), including interstitial and replacement fibrosis using CMR. In a study of 50 patients, with reduced EF, late gadolinium enhancement (a technique which identifies replacement fibrosis) was correlated with a lower septal E/e′ ratio than patients without a scar (*P* = 0.05).

Measurement of dimensions and flow are evaluated similarly to echocardiographic measures, although volumes are more accurate. In tagging, the myocardium is labeled using selective saturation prepulses in specific myocardial regions perpendicular to the imaging plane. This allows strain and 3-D motion analysis (48).

## Cellular Changes with Aging

Increased ventricular stiffness and a change in the microscopic structure of the myocardium are inevitable parts of aging. This, coupled with changes in vascular stiffness, may lead to increased vulnerability in certain groups to developing symptomatic HF (1). Traditional assessment of systolic heart function will not identify these patients. The increased stiffness of the myocardium is thought to be due to changes in the collagen content of the extracellular matrix and increased fibrosis (49). There are other changes including reduced phosphorylation of sarcomeric proteins (50) and changes in Titin (51), which may be of importance at a cellular level.

## Congenital Heart Disease

### Overview

There are five main classes of CHD, which affect diastolic function. Pressure–overload lesions such as aortic stenosis and systemic hypertension cause a decrease in compliance due to hypertrophy. Volume overload leads to increased compliance up until a point, when hypertrophy or fibrosis occurs. Mixed pressure and volume overload can combine to affect compliance, such as in repaired Tetralogy of Fallot with some pulmonary valve stenosis and incompetence. Primary or secondary myocardial diseases can decrease compliance directly, such as in amyloidosis or restrictive cardiomyopathy. Transposition of the great arteries leads to a special situation in which the RV is faced with increased afterload and the LV with a much lower pressure than normal, both of which may cause decreased compliance (52). A summary of lesions and the changes in various parameters used to quantify DD is provided in Table 1.

**Table 1:** **Findings in various congenital cardiac lesions with the onset of diastolic dysfunction**.

Lesion	LA size	Mitral inflow	TDI
HcM	Increased	Increased E/A ratio, DT reduced	E/e′ increased

Aortic stenosis	Increased/normal	DT reduced, shortened A wave duration	E′, A′ reduced, E′ increased

Aortic regurgitation	Enlarged	Increased E/A	MV e′ decreased, E/e′ increased

Mitral stenosis	Enlarged	Low transmitral gradient, short DT	IVRT–T_E-e_′ decreased, E/e′ increased

Mitral regurgitation	Enlarged	Increased A wave reversal velocity	Decreased IVRT–T_E-e′_

Tetralogy of Fallot^a^	Normal	Reduced E/A ratio, shorter IVRT	Reduced MV e′ and a′, reduced TV s′ and e′, increased TV a′

Single ventricle	Normal/enlarged (dependent on anatomy)	MV E decreased	E/e′ increased

*IVRT, isovolumic relaxation time; DT, e-wave deceleration time; E, early left ventricular (LV) filling velocity; A, velocity of LV filling contributed by atrial contraction; s′, myocardial velocity during systole; e′, myocardial velocity during early filling; a′, myocardial velocity during filling produced by atrial contraction; $T_{E-e^{\prime }}$, time interval from onset of E wave to e′; LA, left atrium; MV, mitral valve; TDI, tissue Doppler imaging*.

*^a^End-diastolic forward flow in PA and reduced pulmonary regurgitation*.

## Tetralogy of Fallot

As with the left ventricle, the right ventricle can become stiff and restrictive due to hypertrophy. In Tetralogy of Fallot, the RV becomes a stiff conduit due to RV outflow tract obstruction, with poor diastolic function. This is evidenced by anterograde pulmonary flow with atrial contraction (end-diastolic forward flow in the PA). The presence of DD is a marker of poor short-term surgical outcome. In a study of 50 children with TOF (mean age 5.0 years), 24 had restrictive RV physiology as described. This correlated with lower E/A ratio and IVRT duration. DD also correlated with prolonged intensive care unit stay, longer duration of ionotropic support, and higher doses of diuretics. It was more commonly seen in patients after transannular patch repair (53). In a study of 112 patients, 50 were found to have a restrictive RV. This was associated with larger RV dimensions and RA dimensions, and increased LA length and LA indexed volume on echocardiography (54). There appeared to be a bigger effect on late filling than early filling of the LV and RV restriction appeared to affect LV filling and diastole (decreasing filling and increasing diastolic pressures). This may be due to mechanical effects of the RV on LV filling or increased fibrosis of the LV. Interestingly, children with restrictive RV have an increased RV volume, whereas adults have a reduced volume (55–57). After the initial period, restrictive RV physiology appears to be protective, with decreased duration of pulmonary regurgitation and better maximum oxygen uptake seen in patients with restriction (58).

## Single Ventricle

The Fontan procedure suddenly offloads a previously volume-overloaded ventricle. There is evidence that even in patients with normal systolic function, diastolic function is impaired (59). This is correlated with the length of time since the Fontan operation and decreases over time. Both left and right systemic single ventricles appear to experience systolic and DD, although systemic right ventricular function is more depressed (60). This may be due to adverse remodeling of the right ventricle, which is being exposed to an abnormally high afterload. A study of 28 patients showed a significant correlation between LV filling pressures (measured by E/e′ ratio) and ventilatory efficiency (VE/VO_2_ slope) (*r* = 0.93; *P* < 0.01) (61). This is interesting as parameters of CPET are correlated with hospitalization in Fontan patients (62).

## Atrial Septal Defect (ASD)

In the presence of an ASD with normal pulmonary pressures, the left–right shunt results in reduced LV filling and SV, reduced tissue perfusion, fluid accumulation, increased RV volume, greater RV SV, and finally normalization of LV filling and SV (63). In a small study of patients (*n* = 18) undergoing percutaneous device closure of their ASD, there were no significant changes in TDI and Doppler M-mode indices of diastolic function after closure. However, E wave velocity and E/e′ ratio at the MV annulus did increase significantly, thereby suggesting they are more load-dependent parameters (64). In children with ASD, TDI velocities do not change immediately after device closure. There is also no indication of elevation of left heart filling pressures after device closure in children, suggesting that children are able to accept increased preload and preserve diastolic function (65). This has implications on timing of closure, suggesting that earlier closure is beneficial.

## Aortic Stenosis

In a study of patients with aortic valve disease (8–39 years), those with aortic stenosis or mixed disease were found to have DD, which was related to the degree of left ventricular hypertrophy (LVH). E/e′ correlated with LV end-diastolic pressure on catheterization. DD was found in 37% of all patients in the study, consisting of 37% of those with AS and 47% of those with mixed valve disease (66). Increased chamber stiffness is related to an increased LV mass/EDV ratio. Patients with aortic stenosis have increased interstitial fibrosis, which is related to a worse prognosis (67) and is known to be related to increased chamber stiffness. Secondary pulmonary hypertension may occur due to DD (68).

Cardiac magnetic resonance has a role in functional assessment of the LV in patients with AS. It is also used in tissue characterization and can quantify the degree of interstitial fibrosis and replacement fibrosis (69). There is some evidence that the degree of fibrosis can predict surgical outcome (67).

## Aortic Regurgitation

An incompetent aortic valve increases the end-diastolic volume of the left ventricle. The LV remodels to cope with this extra volume and becomes more compliant, so that diastolic pressures remain normal. Over time, decompensation may occur, in which case diastolic pressures increase as the LV loses the ability to compensate further (70). This is due to increased myocyte cellular diameter and fibrous content of the myocardium (71).

In aortic regurgitation with normal diastolic function, MV inflow consists of predominant E wave filling and annular e′ is increased or normal due to increased LV SV. However, E/e′ is not increased and PA pressures are normal. With the onset of DD (DD), LV filling pressures are elevated on exercise initially and then at rest.

## Mitral Stenosis

Mitral stenosis results in increased LV filling pressure and reduced LV filling due to the restriction of blood flow through the MV. Most patients have normal intrinsic systolic and diastolic myocardial function. Some studies have found DD using conductance catheters, which are independent of loading conditions and acutely reversed after balloon valvuloplasty (72). The mechanism for this is not clear and may be due to restriction of LV relaxation by an immobile and thickened MV.

## Mitral Regurgitation

In isolated MR, LV compliance usually decreases as it dilates to accommodate an increased volume (73). In acute MR, increased LV diastolic pressure is due to increased LV dilatation and a shift upward on the pressure–volume relationship. Chronic MR often leads to remodeling and LV dilatation, thereby retaining LV SV (74).

MR, in the absence of LV DD, will cause an increase in E/A ratio (>1) and increase in transmitral E velocity (75). The e′ velocity is also increased, and E/e′ is not indicative of filling pressures (76). However, A reversal wave velocity does relate to LV diastolic pressures independently of MR (77). The ratio of IVRT to *T*_E–e′_ can also be used to assess diastolic function irrespective of MR (77).

## Cardiomyopathies

### HF with Preserved Ejection Fraction (HFpEF)

One theory of the mechanism of HFpEF is that it is caused by DD. Increased LV filling pressures cause back pressure on the pulmonary circulation, leading to symptoms of HF, including breathlessness. This is assumed to be the case as EF remains in the normal range, which is thought to denote normal systolic function (78). However, there have been studies which show that symptoms of HF in these patients correlate with left ventricular end-diastolic volume and that the SV is only maintained due to LV dilatation. The mechanism postulated is that of excessive LV diastolic dilatation by fiber slippage and creep (79).

The process of LV remodeling to compensate for decreased systolic function in these patients occurs due to feedback from the periphery, causing the heart to adapt with an increase in volume to maintain SV (80). Therefore, an EF of 20% in a dilated ventricle may produce the same SV of a normally sized ventricle with a normal EF (81). Patients with LVH manage to avoid this excessive distension and may be more prone to HF with reduced EF (76). Symptoms of HF, such as breathlessness on exertion, are not related to PCWP (82) or systolic function (83). Instead, the determinants appear to be musculoskeletal status, body composition, motivation, and tolerance of discomfort (82). Therefore, using symptoms alone to determine whether a patient has HF may not be valid.

A number of problems with the definition of HFpEF have been highlighted above; HF may not be reliably diagnosed using symptoms alone, and a preserved EF does not always correlate with normal systolic function. The notion of this type of disease being the definitive model for DD is flawed.

### Hypertrophic Cardiomyopathy

Diastolic dysfunction is well recognized in hypertrophic cardiomyopathy, due to the active component of actin–myosin dissociation in the early filling phase and the passive compliance of the left ventricle (84–87). DD causes a reduced rate and magnitude of LV filling and reduced SV. This results in elevation of LV end-diastolic pressures leading to symptoms of HF. Evaluation of diastolic function is similar to other conditions, with values and ratios changing with severity as expected. In some patients, a restrictive phenotype is present, characterized by increased mitral inflow E/A ratio, reduced DT, and increased pulmonary vein A reversal wave velocity (88). TDI may help to distinguish mild disease in the seemingly normal hearts of disease-causing gene carriers (89, 90).

## Conclusion

Diastolic dysfunction is a characteristic of many types of congenital heart disease as well as of infancy and the aging heart. Thorough and thoughtful evaluation of diastolic function can help to explain symptoms and affect the treatment of patients with seemingly normal ventricular function. Ventricular function should be thought of as a combination of ventricular filling as well as systolic ejection so that the contribution of ventricular compliance to overall heart function is taken into account. Finally, loading conditions and effects of exertion should be taken into account during evaluation.

## Author Contributions

Literature search and writing initial draft: DP. Critical revision and editing: MB. Final draft: DP.

## Conflict of Interest Statement

The authors declare that the research was conducted in the absence of any commercial or financial relationships that could be construed as a potential conflict of interest.
